# Correction: In vitro hepatic metabolism of mefloquine using microsomes from cats, dogs and the common brush-tailed possum (*Trichosurus vulpecula*)

**DOI:** 10.1371/journal.pone.0233223

**Published:** 2020-05-08

**Authors:** The PLOS ONE Staff

## Notice of republication

An incorrect version of Fig 1 was published in error. The publisher apologizes for the error. This article was republished on April 24, 2020, to correct for this error. Please download this article again to view the correct version.
